# Editorial: Recent advances in plant genetic engineering and innovative applications

**DOI:** 10.3389/fpls.2022.1045417

**Published:** 2022-10-19

**Authors:** Ruslan Kalendar, Vladimir Orbovic, Marcos Egea-Cortines, Guo-qing Song

**Affiliations:** ^1^Helsinki Institute of Life Science HiLIFE, University of Helsinki, Helsinki, Finland; ^2^National Laboratory Astana, Nazarbayev University, Astana, Kazakhstan; ^3^University of Florida, Institute of Food and Agricultural Sciences, Lake Alfred, FL, United States; ^4^Universidad Politécnica de Cartagena, Cartagena, Spain; ^5^Plant Biotechnology Resource and Outreach Center, Department of Horticulture, Michigan State University, East Lansing, MI, United States

**Keywords:** plant genetic engineering, transformation, recalcitrant species, genome/gene editing, virus-induced silencing (VIGS), plant artificial minichromosomes, protein-domain specific technology

Plant genetic engineering is one of the most popular advances in plant science and has in recent years become part of mainstream discussion in society (Mackelprang and Lemaux, 2020). This is born out of multiple unresolved debates on genetically modified organisms (GMOs), the involvement of policymakers in an attempt to regulate and ensure its safe application to food crop production, and it also being featured as a topic in the politics of global aid (Steinwand and Ronald, 2020). Apart from the GMO debate, however, several advances in genetic engineering have been recorded that empower plant scientists to investigate and solve previously unexplored problems (Evanega et al., 2022).

From the challenge of transforming recalcitrant species and the development of genetic engineering techniques suitable for non-angiosperms to the development of novel genetic engineering technologies and updates to existing technologies, the field of plant genetic engineering is growing and extending the limits of possibilities in Plant Sciences (Zhang et al., 2019). The extension of plant science research capabilities is particularly important as Plant Science plays a significant role in global hot topics such as climate change and sustainability. The goal of this Research Topic is to highlight studies that embody these advances in new biotechnological tools (NBTs) development and the innovative applications of plant genetic engineering. Studies that focus on NBT development for recalcitrant or previous non-transformable species to allow the unlocking of the biology of these species are of significant interest to this collection. Furthermore, the application of novel strategies of next-generation genetic engineering technologies such as genome/gene editing and protein-domain specific technology (*e.g*., K-Domain technology) (Song and Han, 2021) and innovative applications as well as updates on well-established genetic engineering technologies (*e.g.*, plant artificial minichromosomes and virus-induced silencing) (Yu et al., 2016; Courdavault et al., 2020) will be explored in this collection. Finally, we sought studies that involved the innovative combination of artificial intelligence or machine learning with genetic engineering to investigate (Alley et al., 2020), solve problems, and innovate in plant science.

## Genome editing and transgenic plant technology

Transgenic plant expression of pesticidal proteins derived from *Bacillus thuringiensis* (Bt) and other bacteria has been successfully used for insect pest control. In a paper by Ravanfar et al., transgenic curry tree (*Bergera koenigii*) was produced by expressing the pesticidal protein Cry1Ba1 derived from *Bacillus thuringiensis*. Interestingly, this transgenic can be produced for potential use as trap plants for the suppression of Asian citrus psyllid (*Diaphorina citri*) populations toward the protection of citrus groves from citrus greening.

Another application of using transgenic plants utilizing the K-domain technology to increase maize yield was presented by Song and Han. Keratin-like (K) domain is a conserved protein domain of tens of MIKC-type MADS-box genes in plants. K-domain technology utilizes the expression of the K-domain of a blueberry *SUPPRESSOR OF OVEREXPRESSION OF CONSTANS 1* to regulate plant growth and resulted in grain yield increase by 13 to 100% under different experimental conditions. This K-domain technology opens a new approach to increasing crop yield through its potential to mimic the K-domains of multiple MADS-box genes.

In plants, non-transgenic crop breeding with high production in diverse plants is presented by virus-induced gene silencing (VIGS) genetic tools. VIGS exploits an RNA-mediated antiviral defense mechanism [post-transcriptional gene silencing (PTGS)] for functional gene analysis to fulfill some of this promise in diverse aspects. The plant’s natural defense mechanism is induced by a virus infection, and some endogenous genes that are homologous to viral genomes could also be silenced at the same time. Modification of the viral genome into a recombinant viral vector containing sequence homologous to host genes causes homologous silencing of endogenous genes in plants. The review by Shi et al., summarizes the recent applications in diverse plant species, thus providing a better understanding and advice for functional gene analysis related to crop improvements. As the viral vector construction of tobacco rattle virus (TRV) is an important factor of an efficient VIGS system, many modifications have been undertaken based on the original TRV vector to obtain better silencing efficiency in different species. The development of the VIGS system is strongly affected by the selection of Agrobacterium strain, inoculum concentration, environmental factors, and proper positive controls. All improvements to VIGS technology speed up the application of this tool for identifying the candidate genes involved in various aspects of plant biology), including plant-environment interactions, plant growth and development, metabolic processes, and other cellular processes in plants.

The Brome mosaic virus (BMV)-based VIGS vector involved the establishment of a simple and effective VIGS procedure by Wang et al., in bread wheat genes using PHYTOENE DESATURASE (TaPDS) and PHOSPHATE2 (TaPHO2) as targets. Smaller inserts (~100 nucleotides) were more stable and conferred higher silencing efficiency and longer silencing duration, compared with larger inserts. This VIGS genetic technology has a high potential for a rapid and effective functional genomics tool for high-throughput gene function studies in aerial and root tissues and many plant species.

The application of CRISPR/Cas9 was used for efficient multi-site genome editing in coniferous species (*Picea glauca*) by Cui et al., for target traits modification needed to speed up breeding. This CRISPR/Cas9 system based on somatic embryogenesis was proven for conifers and was optimized based on codon bias in white spruce and a spruce U6 promoter. This conifer-specific CRISPR/Cas9 system was used for multi-site genome editing to target the gene encodes 1-deoxyxylulose 5-phosphate synthase of white spruce by *Agrobacterium-*mediated transformation and is valuable in gene function research and trait improvement.

## Establishment of transgenic root in *Agrobacterium*-mediated gene transformation

The optimization of plant *in vitro* studies, by considering all the influential factors, is laborious, time-consuming, and challenging because of its multifactorial nature. The developed protocols are based on the model for efficiency in predicting the gene transformation efficiency of plants with a low rate of transformation. The multilayer perceptron topology of an artificial neural network (ANN) was applied to create two predictor models in *Agrobacterium*-mediated gene transformation of tobacco by Niedbała. Through precise and efficient data interpretation, ANN could help optimize the gene transformation conditions in *Agrobacterium*-mediated gene transformation studies. A study by Nguyen and. Searle developed a simple, efficient and rapid hairy root transformation system for common vetch to facilitate functional gene analysis. The authors show that the infection of the hypocotyls on 5-day-old *in vitro* or *in vivo*, soil-grown seedlings with *Rhizobium rhizogenes* using a stabbing method on vetch explants was effective at producing transgenics in shoot and hypocotyl-epicotyl explants. This simple method also produces contaminant-free transgenic hairy roots for downstream study and shoots could be helpful in plant propagation.

## Artificial minichromosomes technology

Artificial minichromosomes are the next-generation technology for plant genetic engineering and represent an independent platform for expressing foreign genes and tools for studying the structure and function of chromosomes. This technology for telomere-associated chromosome truncation has been applied successfully in mammals and plants and used as an independent platform for stacking multiple foreign genes without gene segregation. The truncated minichromosome was employed as a platform to receive foreign genes in *Brassica nap*us by Yin et al.. This research primarily focused on the development of stably inherited minichromosomes and their precise characterization and tracking over different generations. A 0.35-kb direct repeat of the Arabidopsis telomeric sequence was transformed into *Brassica napus* to produce artificial minichromosomes, which were analyzed by multifluorescence *in situ* hybridization (multi-FISH), genome resequencing, and insertion of site-specific PCR, primer extension telomere rapid amplification (PETRA).

In summary, the studies collected on this Research Topic reveal advances in next generation sequencing and gene editing technologies that have revolutionized plant science research and empowers plant biotechnologists to manipulate target gene(s) more precisely and effectively in genetic engineering. We believe that a breakthrough in plant genetic engineering is being made and is going to drive a second Green Revolution that is the key to feeding our future.

## Author contributions

RK prepared the draft. All authors listed have made a substantial, direct, and intellectual contribution to the work and have approved it for publication.

## Funding

This work was supported by the Science Committee of the Ministry of Education and Science of the Republic of Kazakhstan (AP14869076) to RK.

## Acknowledgments

We thank all authors and reviewers for their contributions to this Research Topic and the support of the editorial office.

## Conflict of interest

The authors declare that the research was conducted in the absence of any commercial or financial relationships that could be construed as a potential conflict of interest.

## Publisher’s note

All claims expressed in this article are solely those of the authors and do not necessarily represent those of their affiliated organizations, or those of the publisher, the editors and the reviewers. Any product that may be evaluated in this article, or claim that may be made by its manufacturer, is not guaranteed or endorsed by the publisher.
